# Functional psychosurgery in psychiatry: indications and preoperative evaluation. A narrative review

**DOI:** 10.3389/fpsyt.2026.1844401

**Published:** 2026-08-19

**Authors:** Álvaro Moleón-Ruiz, Paloma Álvarez de Toledo, Manuela Martín-Bejarano, Miquel Bioque, Cristina V. Torres Díaz

**Affiliations:** 1 Instituto Andaluz de Salud Cerebral, Seville, Spain; 2 Psychiatry Service, Hospital Virgen del Rocío, Seville, Spain; 3 Barcelona Clínic Schizophrenia Unit, Institute of Neurosciences, Hospital Clinic of Barcelona, IDIBAPS, CIBERSAM, University of Barcelona, Barcelona, Spain; 4 Department of Neurosurgery, Hospital Universitario de La Princesa, Madrid, Spain

**Keywords:** circuit-informed assessment, deep brain stimulation, functional psychosurgery, neuropsychological evaluation, patient characterization, preoperative assessment, treatment-resistant psychiatric disorders

## Abstract

Functional neurosurgery has evolved from a marginal and controversial intervention into a promising, technically feasible option for carefully selected patients with treatment-resistant psychiatric disorders. Among available techniques, deep brain stimulation has gained increasing clinical acceptance for selected patients with severe obsessive–compulsive disorder and Tourette syndrome, while other psychiatric indications, including treatment-resistant depression, substance use disorders, schizophrenia, anorexia nervosa and pathological aggression, remain largely investigational or restricted to clinical trials and highly specialized programs. However, the absence of harmonized preoperative assessment standards across centers highlights the need for a structured, multidisciplinary framework to guide consistent clinical decision-making and ensure procedural safety. The framework proposed in this review is intended to support structured clinical profiling and outcome monitoring, but should not be interpreted as a substitute for professional consensus recommendations, multidisciplinary clinical judgment, ethics committee review, or applicable regulatory requirements. This narrative review examines the current role of functional psychosurgery in psychiatry, with particular focus on structured preoperative assessment models that optimize patient characterization within specialized clinical settings. We propose a pragmatic, circuit-informed framework integrating diagnostic precision, psychopathological assessment, neuropsychological profiling, functional evaluation, and ethical-regulatory oversight. By synthesizing evidence across psychiatric disorders, neurobiological models, and multidimensional assessment strategies, we outline a practical framework for preoperative characterization and outcome monitoring in psychiatric neurosurgery. When applied within specialized centers and under rigorous clinical, ethical, and regulatory oversight, functional psychosurgery may offer clinically meaningful symptom relief and improved quality of life for carefully selected patients who have exhausted conventional treatments.

## Introduction

Severe, treatment-resistant psychiatric disorders remain one of the most demanding challenges in contemporary medicine, exerting a profound toll on quality of life, functional capacity, and mortality despite advances in pharmacotherapy and psychotherapy (1, 2). For a considerable proportion of patients, debilitating symptoms persist, prompting the search for alternative strategies and paving the way for the development of neurosurgical approaches in psychiatry.

Given the substantial variability in regulatory frameworks, reimbursement policies, and clinical implementation across jurisdictions, this review is primarily intended for clinicians working within European healthcare systems. In this context, DBS for psychiatric disorders remains restricted to a limited number of indications and highly specialized centers, with substantial differences between established and investigational applications.

Among these alternatives, functional psychosurgery, particularly deep brain stimulation (DBS), has emerged as a promising intervention for selected psychiatric disorders. However, the level of clinical evidence and regulatory acceptance differs substantially across indications. DBS has undergone a striking transformation, evolving from a last-resort option into a therapeutic tool increasingly supported by a growing understanding of brain networks and governed by strict ethical and clinical criteria (2, 3). In this context, the surgical aim is not complete cure, an unrealistic expectation in most cases, but rather a meaningful reduction in suffering and functional impairment, enabling a substantial improvement in the patient’s quality of life (1, 2, 4).

Building on this conceptual framework, contemporary psychiatric neurosurgery increasingly recognizes psychiatric disorders as disorders of distributed brain circuits rather than isolated anatomical abnormalities (5). Converging evidence from neuroimaging, lesion studies, and neurophysiology has identified dysfunction within cortico-striato-thalamo-cortical, reward, salience, and frontolimbic networks across several treatment-resistant psychiatric disorders. These neurobiological models provide a useful framework for understanding symptom dimensions and may help guide the selection of clinically relevant domains for preoperative and postoperative assessment. Although these circuit models are increasingly used to understand disease mechanisms, their greatest clinical utility may currently lie in informing patient characterization rather than target selection (6, 7). Different neural networks give rise to distinct psychopathological, cognitive, and functional symptom dimensions, which should be systematically evaluated before and after intervention to ensure a comprehensive assessment of treatment outcomes.

This network-based approach has been greatly enhanced by progress in our neurobiological understanding, while advances in neuromodulation technology have enabled highly selective interventions in brain circuits implicated in the genesis and persistence of psychiatric symptoms (1, 5). Among psychiatric indications, obsessive–compulsive disorder (OCD) currently represents the most established application of DBS, supported by the largest body of evidence, the longest clinical experience, and the most clearly defined candidate selection criteria. Accordingly, DBS may be considered in carefully selected patients with severe, chronic, and treatment-resistant OCD who remain significantly impaired despite optimized pharmacological and psychotherapeutic interventions (1–3). In February 2009, the U.S. Food and Drug Administration (FDA) granted a Humanitarian Device Exemption (HDE) for DBS in OCD, marking a major milestone in the clinical development of psychiatric neurosurgery (9).

Tourette syndrome (TS) has also accumulated a growing evidence base and is increasingly considered a therapeutic option in highly refractory cases treated within specialized centers, particularly when severe tics persist despite optimized pharmacological and behavioral interventions, although regulatory pathways remain heterogeneous (11, 12).

Treatment-resistant depression (TRD) represents one of the most extensively investigated experimental indications for psychiatric DBS. Although several clinical trials have reported encouraging outcomes, evidence remains heterogeneous and DBS for depression is generally restricted to research protocols and highly specialized centers (1, 2, 10).

Other emerging applications include substance use disorders (SUD), schizophrenia (SCZ), anorexia nervosa (AN), and pathological aggression (PA). In these conditions, current evidence derives primarily from clinical trials, case series, and compassionate-use experiences, suggesting potential benefit in carefully selected treatment-refractory patients while remaining insufficient to support routine clinical implementation (13–23).

Taken together, substantial differences in evidence quality, regulatory status, and clinical experience persist across indications. While OCD currently represents the psychiatric indication with the strongest clinical evidence and most clearly defined regulatory pathway, the remaining conditions discussed in this review exist along a spectrum of investigational maturity, with varying levels of evidence, regulatory acceptance, and clinical implementation. These differences reinforce the need for rigorous patient characterization and standardized assessment procedures.

Although the level of evidence and regulatory status differ substantially across psychiatric indications, the principles of comprehensive preoperative assessment described in this review are applicable to both established and investigational uses of psychiatric neurosurgery, where rigorous patient characterization and outcome monitoring are equally essential. However, the clinical applicability of the proposed framework is currently strongest for indications with the most established clinical experience, particularly OCD, whereas its use in highly investigational conditions should be interpreted more cautiously given the limited evidence base. For these indications, any assessment recommendations should be considered exploratory and applied within appropriate research, compassionate-use, or institutionally approved frameworks subject to ethical oversight.

Although these disorders differ substantially in their clinical presentation, converging evidence suggests that many arise from dysfunction within distributed brain networks involving affective, cognitive, reward, salience, and cortico-striato-thalamo-cortical circuits. Consequently, preoperative assessment should extend beyond diagnostic confirmation and symptom severity, incorporating the psychopathological, cognitive, and functional dimensions most closely linked to the underlying neurobiological dysfunction (5–7).

From a clinical perspective, the value of these neurobiological models lies in their ability to contextualize symptom expression across multiple domains. By linking disorder-specific circuit dysfunction to psychopathological, cognitive, and functional manifestations, they provide a conceptual framework for comprehensive patient characterization and for the systematic evaluation of outcomes following intervention (6, 7).

Despite increasing interest in psychiatric neuromodulation, there is currently no harmonized clinical framework to guide multidisciplinary decision-making and patient selection across European settings. Variability in regulatory policies and access further underscores the need for structured evaluation models grounded in ethical and clinical standards.

The aim of this narrative review is to synthesize current evidence regarding clinical indications, multidisciplinary preoperative assessment, and neurobiologically informed characterization of candidates for psychiatric neurosurgery. We seek to provide a practical framework that links disorder-specific circuit dysfunction to the psychopathological, neuropsychological, and functional domains most relevant for baseline characterization and postoperative outcome monitoring. The framework proposed should therefore be regarded as a complementary tool to support structured assessment and outcome monitoring rather than as a substitute for professional consensus recommendations, multidisciplinary clinical judgment, or ethical and regulatory oversight.

## Criteria for considering psychosurgery

A fundamental prerequisite is the establishment of a clear, stable diagnosis, defined according to DSM-5 or ICD-11 criteria, and belonging to a psychiatric disorder with sufficient scientific evidence supporting potential benefits from psychosurgical intervention. Clinical refractoriness must be well documented and typically includes the following (1–3):

- Failure of three or more optimized pharmacological treatments, including appropriate combination or augmentation strategies (e.g., antidepressants with atypical antipsychotics or glutamatergic modulators for depression).
- Completion of at least one structured psychotherapeutic intervention, grounded in evidence-based models such as cognitive-behavioral therapy tailored to the specific disorder.
- An illness duration of five years or more, accompanied by persistent, severe functional impairment, verified through validated scales (see *Functional evaluation* section).

These core requirements are applicable across all psychiatric diagnoses considered for functional neurosurgery. **Table 1** summarizes the disorder-specific eligibility criteria derived from the requirements most consistently reported across published DBS studies and expert recommendations for each indication. Given the heterogeneity of the available evidence, these criteria should be regarded as a pragmatic framework to support multidisciplinary decision-making rather than as universally accepted eligibility requirements (2, 11, 13, 17, 19, 22, 24–26). For better-established indications, they may assist routine clinical evaluation and patient characterization, whereas for investigational indications they should be interpreted within the context of local regulatory requirements, ethics committee oversight, and the specific objectives of the clinical or research program.

**Table 1:** Commonly reported eligibility criteria across psychiatric DBS indications.

Disorder	Specific inclusion criteria
Obsessive–compulsive disorder	Y-BOCS ≥28 after optimized treatment (32); failure of ≥3 SSRIs or serotonergic antidepressants (≥12 weeks each); at least one pharmacological augmentation with an atypical antipsychotic; structured CBT protocol focusing on exposure and response prevention (≥20 sessions).
Depression	Diagnosis of major depressive episode (unipolar or bipolar II); failure of ≥3 antidepressants from different classes (≥6 weeks each); at least one pharmacological augmentation (lithium, atypical antipsychotics, minimum 4–6 weeks); structured evidence-based psychotherapy (typically CBT, ≥12 sessions); sustained MADRS ≥30 (28).
Tourette syndrome	Confirmed diagnosis; severe clinical course with disabling motor and vocal tics despite full treatment (YGTSS ≥35) (33); failure of ≥2 pharmacological treatments for tics (e.g., antipsychotics, tetrabenazine); documented trial of specific behavioral therapy (CBIT/HRT); ≥6 months of psychiatric stability.
Pathological aggression	Diagnosis of intermittent explosive disorder or other disruptive disorder with severe violence; failure of ≥2 antipsychotics and one mood stabilizer at adequate dose/duration; documented behavioral refractoriness with high OAS/MOAS (29, 30) scores; history of need for restraints/institutionalization; multidisciplinary evaluation of surgical feasibility. It should be noted that pathological aggression is not a formal diagnosis but rather a symptom that can manifest across different neuropsychiatric conditions, which has regulatory implications for device approval and clinical indication.
Substance use disorders	Severe SUD (≥5 years, high functional impact); multiple relapses despite ≥3 structured treatments (CBT, community reinforcement programs, appropriate pharmacotherapy); stabilized psychiatric comorbidity; at least one prolonged abstinence period followed by relapse; high scores on addiction severity scales; strong motivation and previous adherence to rehabilitation programs.
Schizophrenia	Schizophrenia diagnosis per DSM-5; ≥2 years of persistent positive or negative symptoms; failure of ≥2 antipsychotics (including clozapine or contraindication); high PANSS (31) score; clinical stability without active psychotic episodes.
Anorexia nervosa	DSM-5 (27) AN diagnosis; ≥10-year illness course; BMI <14 sustained ≥12 months despite specialized care; failure of inpatient and structured psychotherapies; frequent comorbid affective/obsessive symptoms and high life-threatening risk.

CBIT, Comprehensive Behavioral Intervention for Tics; CGI-S, Clinical Global Impression – Severity; DSM-5, Diagnostic and Statistical Manual of Mental Disorders, Fifth Edition; HRT, Habit Reversal Training; BMI, body mass index; SSRI, selective serotonin reuptake inhibitor; MADRS, Montgomery–Åsberg Depression Rating Scale; MOAS, Modified Overt Aggression Scale; OAS, Overt Aggression Scale; PANSS, Positive and Negative Syndrome Scale; CBT, cognitive–behavioral therapy; Y-BOCS, Yale–Brown Obsessive Compulsive Scale; YGTSS, Yale Global Tic Severity Scale.

Criteria were synthesized from eligibility requirements most commonly reported in published DBS studies and expert recommendations. They are intended to support clinical decision-making and may vary across clinical programs, research protocols, and regulatory jurisdictions.

## Risk–benefit assessment

A comprehensive evaluation of potential risks and expected benefits is essential before proceeding with any surgical intervention. Functional neurosurgery should only be considered when there is a realistic likelihood of significant clinical and functional improvement, and when the patient’s overall condition does not present factors that may compromise safety or efficacy (4, 5).

Absolute or relative exclusion criteria include (3):

- Major neurocognitive disorder or progressive neurological disease
- Disorganized or unstable psychotic state
- Severe or uncontrolled medical comorbidities

In addition, the following elements must be systematically assessed:

- The patient’s motivation, treatment adherence, and level of cooperation
- A realistic understanding of potential risks, benefits, and limitations, by both patient and family.
- A prior positive response to electroconvulsive therapy (ECT), as it could indicate higher likelihood of benefit from DBS.
- The availability of a stable support system (e.g., family, housing, consistent clinical follow-up).

Although serious adverse events are relatively uncommon, documented complications may include central nervous system infections, hardware malfunction, and transient mood changes. Postoperative emergence of suicidal ideation or behavioral disorganization has been reported in isolated cases (1, 2, 10), reinforcing the importance of long-term coordinated follow-up and crisis management planning.

## Ethical and regulatory considerations

All psychosurgical procedures must be conducted under strict ethical, legal, and human rights frameworks. Informed consent must be competent, voluntary, and clearly documented, with particular care taken to ensure the patient fully understands the nature of the intervention. In cases where decision-making capacity is uncertain, a structured neuropsychological evaluation should inform the consent process. Moreover, contemporary ethical frameworks increasingly advocate for supported consent approaches and the incorporation of psychiatric advance directives to uphold autonomy and promote equitable access to DBS in refractory psychiatric disorders (34, 35). Regulatory oversight of DBS for psychiatric disorders varies substantially across jurisdictions. In the United States, specific psychiatric indications may be considered through FDA device pathways, including Humanitarian Device Exemption mechanisms. In Europe, DBS is regulated within the medical device framework, and access depends on conformity assessment procedures, national competent authorities, institutional governance, and ethics committee oversight. Consequently, the availability and implementation of psychiatric DBS may differ considerably between countries and healthcare systems.

Ethical considerations must include:

- Respect for autonomy and identity
- Avoidance of coercion or therapeutic misconception
- Consideration of potential impacts on agency, authenticity, and personal narrative

From a regulatory perspective, requirements vary according to jurisdiction and indication. For investigational psychiatric indications, procedures generally require approval from an appropriate Research Ethics Committee (CEIm or equivalent body), and may additionally require authorization from national competent authorities depending on local regulations. In Spain, compassionate or off-label use often requires authorization from the Spanish Agency of Medicines and Medical Devices (AEMPS), though requirements may vary by autonomous region. In addition, financial coverage remains uneven across regions, as only some public health systems include selected DBS interventions for psychiatric indications under exceptional or limited-access programs.

The regulatory status varies by disorder:

- OCD: DBS is approved in the U.S. under a Humanitarian Device Exemption (HDE #H050003) since 2009. However, access remains limited due to insurance restrictions and classification as experimental. In Europe, DBS for OCD initially obtained CE marking in 2009, but this certification has not been maintained under current regulatory frameworks, leading to progressively restricted clinical availability. In practice, its use is confined to highly specialized centers or compassionate-use programs, with regional variability in reimbursement and institutional coverage.
- TRD and SUD: DBS has no established psychiatric indication comparable to OCD within major regulatory frameworks. In the United States, its use remains largely restricted to research protocols and investigational pathways. In Europe, access depends on local regulatory requirements, ethics committee approval, institutional policies, and country-specific research or compassionate-use frameworks.
- Tourette syndrome: regulatory pathways remain heterogeneous across jurisdictions. In Europe, DBS generally remains an off-label intervention, with access depending on local regulatory requirements, institutional policies, and reimbursement pathways.
- PA, SCZ, and AN: No formal regulatory approval exists. All procedures must be framed as compassionate use, requiring ethics committee and (when applicable) agency approval, with consent by the patient or legal representative.

In all cases, psychosurgery should be embedded in a comprehensive longitudinal care plan, with robust clinical and psychosocial support. Final eligibility must be determined by a multidisciplinary team, integrating clinical, neuropsychological, functional, and ethical dimensions, and based on a structured preoperative evaluation that includes both transdiagnostic and disorder-specific criteria (4, 36). Consequently, the practical implications of the framework proposed in this review are most directly applicable to better-established indications, whereas its application in investigational disorders should be considered within the context of research, compassionate-use programs, and evolving regulatory frameworks.

## Neurobiological circuits and clinical assessment domains

Although target selection remains a critical component of psychiatric neurosurgery, it is beyond the scope of this review to provide target-selection algorithms. Instead, understanding the neurobiological circuits most consistently implicated in each disorder may help clinicians identify the psychopathological, cognitive, and functional domains that should be systematically assessed before and after intervention (5–7).

**Table 2** summarizes the main neurosurgical targets currently employed in the treatment of refractory psychiatric disorders, outlining their preferred clinical indications and key therapeutic outcomes reported in recent literature (4, 8).

**Table 2:** Main neurosurgical targets used in refractory psychiatric disorders, with preferred clinical indications and evidence of therapeutic response.

Disorder	Main targets	Preferred clinical indications	Key evidence
Obsessive–compulsive disorder	VC/VS, NAc, STN, ITP	Severe resistance; pure or mixed obsessive–compulsive profile	VC/VS: 50–70% Y-BOCS (32) response; ITP: up to 100% response in pilot series (1, 37).
Depression	sgACC, VC/VS, NAc, MFB	Persistent affective symptoms, anhedonia, anxious comorbidity	Remission rates of 34–37% at 12 months (38); MFB: rapid and sustained response (39).
Tourette syndrome	GPi, CM-Pf	Disabling motor/vocal tics; comorbid OCD/ADHD	GPi: −58% YGTSS (33), improvement in BDI (45) and quality of life (12, 40).
Pathological aggression	PMH, VC/VS, NAc, Amygdala	Refractory violent behavior, neurodevelopmental or affective comorbidity	PMH: sustained improvement in 5/7 patients; tractography implicates VTA, dorsal longitudinal fasciculus, and medial forebrain bundle (41). VC/VS: −63% OAS (16, 30); Amygdala: improvement in 70% (14).
Substance use disorders	NAc, MFB, VTA, VC/VS	Persistent craving, multiple relapses, affective comorbidity	Reduced craving and maintained abstinence (17, 18).
Schizophrenia	NAc, sgACC, Habenula, SNpr, VC/VS	Resistant negative, affective, or positive symptoms; anhedonia	Improvement in 57% of patients; significant PANSS (31) reduction; cortical functional activation and network modulation of frontolimbic circuits (19, 20, 42).
Anorexia nervosa	NAc, sgACC, VC/VS, VTA, lateral hypothalamus	BMI <14 sustained; affective symptoms and motivational dysfunction	~60% response, +3.4 BMI points, reduced craving and emotional reactivity (21, 22, 43, 44).

VC/VS, ventral capsule/ventral striatum; NAc, nucleus accumbens; STN, subthalamic nucleus; ITP, inferior thalamic peduncle; sgACC, subgenual anterior cingulate cortex; MFB, medial forebrain bundle; GPi, globus pallidus internus; CM-Pf, centromedian–parafascicular complex; PMH, posterior medial hypothalamus; VTA, ventral tegmental area; SNpr, substantia nigra pars reticulata; BMI, body mass index; Y-BOCS, Yale–Brown Obsessive Compulsive Scale; YGTSS, Yale Global Tic Severity Scale; BDI, Beck Depression Inventory; OAS, Overt Aggression Scale; PANSS, Positive and Negative Syndrome Scale.

Across disorders, the ventral capsule/ventral striatum (VC/VS), nucleus accumbens (NAc), and subgenual anterior cingulate cortex (sgACC) appear as common hubs of intervention, reflecting their central role in affect regulation, motivation, and compulsivity. Other targets, such as the medial forebrain bundle (MFB), posteromedial hypothalamus (PMH), and globus pallidus internus (GPi), have shown utility in modulating specific symptom dimensions (e.g., aggression, anhedonia, tics).

Notably, long-term follow-up in patients with refractory pathological aggression suggests that PMH stimulation can lead to sustained behavioral improvement. Tractographic studies indicate that its therapeutic effects may involve downstream modulation of the ventral tegmental area (VTA), dorsal longitudinal fasciculus, and the medial forebrain bundle (41). Similarly, evidence from connectomic analyses in autism spectrum disorder and extreme behavioral phenotypes has shown that diverse DBS targets may converge onto a common anatomical network, centered on the anterior limb of the internal capsule, and functionally connected to the amygdala, insula, and anterior cingulate cortex (46).

Taken together, these findings reinforce the view that therapeutic response in psychiatric DBS is better explained by modulation of distributed brain circuits rather than stimulation of isolated structures. Collectively, these observations support the relevance of network-based models for understanding symptom dimensions and structuring multidimensional assessment across disorders. Understanding these networks may help clinicians identify the symptom dimensions most relevant to evaluate before and after intervention, supporting a more comprehensive and standardized assessment framework across disorders (5–7).

## Structured preoperative assessment

Preoperative evaluation in psychiatric neurosurgery must be exhaustive, protocolized, and tailored to the clinical and functional profile of each candidate. This section outlines the essential components of such evaluation, distinguishing between transdiagnostic instruments applicable to all candidates and disorder-specific tools required for nuanced clinical characterization.

The purpose of this multidimensional assessment is not only to establish diagnostic eligibility, but also to provide a comprehensive characterization of the patient across psychopathological, cognitive, functional, and psychosocial domains. The information obtained supports multidisciplinary clinical decision-making, helps identify potential vulnerabilities or contraindications, establishes a robust preoperative baseline, and facilitates the interpretation of postoperative changes over time.

## Psychopathological evaluation

The primary goals of the preoperative psychopathological assessment are to confirm diagnostic stability, quantify the severity and chronicity of the disorder, and document clinical refractoriness (4, 38). This process should combine a structured clinical interview with internationally validated rating scales (2, 3). A rigorous approach ensures precise diagnosis, minimizes the risk of misclassification, and helps rule out comorbidities or alternative diagnoses that may influence surgical outcomes (1, 3).

Core instruments that can be applied across neuropsychiatric disorders include:

- Structured Clinical Interview (e.g., SCID-5) (47) to establish primary diagnosis and comorbidities.
- Clinical Global Impression (CGI-S/I) for assessing overall illness severity and change over time.
- Suicide risk and self-harm/aggression assessment tools (e.g., C-SSRS) (48) when clinically indicated.

In addition to the core instruments applied across diagnoses, the use of disorder-specific assessment tools is crucial to ensure accurate clinical characterization and support multidisciplinary clinical decision-making (**Table 3**). For OCD, it is essential to quantify symptom severity and persistence with precision, while also systematically screening for comorbid depressive, anxiety, or autism spectrum symptoms that may shape prognosis (3, 25). In TRD, the evaluation should focus on the intensity and chronicity of affective symptoms, coupled with a thorough assessment of suicidal ideation and self-harm behaviors, given their critical implications for patient safety and treatment outcomes (2, 10). For TS, the assessment must capture both motor and vocal tics in detail and identify comorbidities such as OCD, ADHD, or mood disorders, as these can affect eligibility for surgery and influence postoperative management (11). In cases of PA, a precise appraisal of the frequency, intensity, and type of aggressive behaviors is required, incorporating collateral information from caregivers or institutional records, as patients often show limited insight into their behavior (13, 26). For SUD, evaluation should detail the pattern and severity of substance use, assess craving intensity, and identify relevant psychiatric comorbidities, particularly affective or psychotic disorders, that may influence both candidacy and therapeutic response (17, 24). In SCZ, assessment should encompass positive, negative, and affective symptom domains, alongside cognitive deficits, to refine patient profiling and guide the selection of optimal surgical targets (19, 49). Finally, for refractory AN, the evaluation must address the severity and duration of dietary restriction, the presence of affective and obsessive comorbidities, the patient’s insight into the illness, and their motivation for change, all factors that can be decisive in predicting surgical outcomes (21, 22).

**Table 3:** Disorder-specific psychopathological assessment tools.

Disorder	Core tests	Recommended tests
Obsessive–compulsive disorder	Y-BOCS (32)	BAI (51)/BDI-II (52)
Depression	MADRS (28)	HDRS (65)/BDI-II/C-SSRS (48)
Tourette syndrome	YGTSS (33)	GTS-QOL (63)/BDI-II/Y-BOCS (32)/CAARS (55)/DIVA-5 (59)/GAD-7 (62)/LSAS (67)/BIS-11 (53)
Pathological aggression	OAS (30)/MOAS (29)	ICAP (66)
Substance use disorders	OCDUS (64)/SDS (69)	TLFB (71)/EuropASI (61)/VAS (72)/SDS/BDI-II/SF-36 (70)/substance-specific scales (AUQ (50), CCQ (56), HCQ (68))
Schizophrenia	PANSS (31)	BNSS (54)/CDSS (57)
Anorexia nervosa	EDE-Q (60)/CIA (58)	Y-BOCS/BDI-II/SF-36

AUQ, Alcohol Urge Questionnaire; BAI, Beck Anxiety Inventory; BDI-II, Beck Depression Inventory-II; BIS-11, Barratt Impulsiveness Scale; BNSS, Brief Negative Symptom Scale; CAARS, Conners’ Adult ADHD Rating Scales; CCQ, Cocaine Craving Questionnaire; CDSS, Calgary Depression Scale for Schizophrenia; CIA, Clinical Impairment Assessment; C-SSRS, Columbia Suicide Severity Rating Scale; DIVA-5, Diagnóstico de Trastorno por Déficit de Atención e Hiperactividad en Adultos – Versión 5.0; EDE-Q, Eating Disorder Examination Questionnaire; EuropASI, European Addiction Severity Index; GAD-7, Generalized Anxiety Disorder 7-item scale; GTS-QOL, Gilles de la Tourette Syndrome–Quality of Life Scale; HCQ, Heroin Craving Questionnaire; HDRS, Hamilton Depression Rating Scale; ICAP, Inventory for Client and Agency Planning; LSAS, Liebowitz Social Anxiety Scale; MADRS, Montgomery–Åsberg Depression Rating Scale; MOAS, Modified Overt Aggression Scale; OAS, Overt Aggression Scale; OCDUS, Obsessive Compulsive Drug Use Scale; PANSS, Positive and Negative Syndrome Scale; SDS, Sheehan Disability Scale; SF-36, Short Form Health Survey 36; TLFB, Timeline Follow-Back; VAS, Visual Analog Scale; Y-BOCS, Yale–Brown Obsessive Compulsive Scale; YGTSS, Yale Global Tic Severity Scale.

It is recommended that this evaluation be conducted over at least two separate visits, with a minimum interval of two weeks, to confirm the persistence of the clinical presentation.

## Neuropsychological evaluation

Neuropsychological assessment is a cornerstone of the preoperative work-up for psychiatric neurosurgery. Its primary objectives are to exclude significant cognitive impairment, identify deficits directly related to the underlying disorder, and establish a reliable baseline for postoperative follow-up (73). A comprehensive evaluation should, at minimum, include the assessment of attentional processes, processing speed, reasoning abilities, and multiple components of executive functioning, such as working memory, inhibitory control, and cognitive flexibility, alongside verbal and visual memory. Depending on the clinical presentation, additional domains such as emotion recognition and theory of mind should be explored, particularly when social or affective disturbances are prominent. The use of a standardized transdiagnostic battery (**Table 4**), applied to all candidates, allows clinicians to detect patterns of cognitive dysfunction that may influence surgical candidacy, guide clinical decision-making, and inform long-term postoperative monitoring (1, 73). The proposed set of instruments is grounded in the attention model of Sohlberg and Mateer and the executive function framework of Tirapu-Ustárroz, complemented by key domains such as memory, reasoning, processing speed, and social cognition to ensure a thorough and comparable assessment across patients (74–76).

**Table 4:** Recommended transdiagnostic neuropsychological battery for preoperative assessment in functional psychiatric neurosurgery.

Cognitive domain (theoretical framework)	Recommended neuropsychological tests
Attention (74)	D2 Test (77), TMT A/B (78), CPT (79), WAIS-IV Digit Span (forward) (80)
Processing speed	Digit Symbol (WAIS-IV), TMT-A
Executive Functions (75)	WCST (81), Stroop Test (inhibitory control) (82), TMT-B (cognitive flexibility), Tower of London (planning) (83), WAIS-IV Digit Span (backward), N-back task (working memory) (84).
Verbal and abstract reasoning	Similarities (WAIS-IV), Matrices (WAIS-IV), Raven’s Progressive Matrices (85).
Verbal and visual memory	CVLT (86) or FCSRT (87); ROCF (88) for visual memory
Social cognition and emotion recognition	Ekman Facial Emotion Recognition Test (89), Social Stories (90), Faux Pas Test (theory of mind) (91).

CPT, Continuous Performance Test; CVLT, California Verbal Learning Test; FCSRT, Free and Cued Selective Reminding Test; ROCF, Rey–Osterrieth Complex Figure; RPM, Raven’s Progressive Matrices; TMT, Trail Making Test; WAIS-IV, Wechsler Adult Intelligence Scale – Fourth Edition; WCST, Wisconsin Card Sorting Test.

This battery ensures the collection of comparable data across all patients, facilitates the identification of deficits that could influence clinical decision-making or identify factors relevant for patient selection and postoperative management, and establishes a baseline profile for long-term monitoring (2, 73).

Beyond this core protocol, disorder-specific neuropsychological tests may be warranted (**Table 5**). The selection of disorder-specific neuropsychological measures should be guided by the cognitive domains most consistently associated with the underlying circuit dysfunction. In obsessive–compulsive disorder, abnormalities within cortico-striato-thalamo-cortical (CSTC) circuits are frequently associated with impaired inhibitory control, cognitive rigidity, and reduced flexibility (8), supporting the inclusion of tasks specifically targeting these domains. In treatment-resistant depression, dysfunction involving the subgenual anterior cingulate cortex, default mode network, and reward-related circuits has been linked to psychomotor slowing, maladaptive affective processing, and executive inefficiency (2), making processing speed and emotional-cognitive measures particularly relevant. In substance use disorders, disruption of mesocorticolimbic reward pathways is associated with impaired decision-making, heightened reward sensitivity, and preference for immediate reinforcement (6, 7), justifying the assessment of risk-based decision-making and delay discounting. In schizophrenia, alterations within salience, frontotemporal, and frontoparietal networks are consistently associated with deficits in working memory, executive functioning, and social cognition (19), supporting the inclusion of targeted measures in these domains. Therefore, disorder-specific neuropsychological assessment should not be viewed as an adjunctive component of the evaluation process, but rather as a neurobiologically informed strategy for characterizing the cognitive dimensions most relevant to each disorder and for monitoring clinically meaningful postoperative change.

**Table 5:** Recommended complementary neuropsychological tests by disorder.

Disorder	Recommended neuropsychological tests
Obsessive–compulsive disorder	Hayling Sentence Completion Test (92), Go/No-Go (93).
Depression	Ekman 60 Faces Test (94), Raven’s Progressive Matrices (85).
Tourette syndrome	Iowa Gambling Task (95), Go/No-Go
Pathological aggression	Functional observation, caregiver interviews, KBIT-2 (96) in cases with intellectual disability
Substance use disorders	Stop Signal Task (97), Delay Discounting Task (98).
Schizophrenia	Theory of mind tasks (99), WAIS-IV Letter–Number Sequencing (80).
Anorexia nervosa	Iowa Gambling Task, Emotional Recognition Test (94).

Go/No-Go, behavioral inhibition task; KBIT-2, Kaufman Brief Intelligence Test, Second Edition; Stop Signal Task, response inhibition task; Delay Discounting Task, delay discounting task; Letter–Number Sequencing, letter–number sequencing (WAIS-IV).

Administration should always be conducted by an experienced clinical neuropsychologist with expertise in psychiatric populations, with results discussed within the multidisciplinary team.

## Functional evaluation

Functional impairment represents the clinical expression of symptom burden and cognitive dysfunction and therefore constitutes a critical outcome domain across all psychiatric indications. Severe and persistent functional impairment is a cross-cutting prerequisite for considering psychiatric neurosurgery, regardless of the specific diagnosis. Functional assessment should be systematic, performed on at least two separate occasions, and enriched with third-party information from relatives, caregivers, or relevant institutional reports (e.g., school or workplace records). Core instruments recommended for all candidates include:

- WHODAS 2.0 (World Health Organization Disability Assessment Schedule, version 2.0) (100): scores above 75 indicate severe global dysfunction.
- Sheehan Disability Scale (SDS) (69): scores ≥7 in at least two domains (work, social, family) denote marked impairment.
- Clinical Global Impression – Severity (CGI-S) (101): scores ≥5 reflect moderate-to-severe symptom burden.

While these tools provide a standardized framework for gauging functional severity, the evaluation must also consider diagnosis-specific functional domains. For example, in Tourette syndrome, the focus is on social, occupational, and academic interference linked to tics and comorbidities; in pathological aggression, on supervision needs, institutionalization, and the use of physical restraints; in substance use disorders, on quality of life, addiction severity, and psychosocial impact; and in refractory anorexia nervosa, on self-care abilities, nutritional intake, and personal autonomy. **Table 6** summarizes these disorder-specific functional domains along with recommended complementary instruments, ensuring that both global and diagnosis-targeted measures are integrated into the preoperative work-up.

**Table 6:** Disorder-specific functional domains and recommended complementary assessment tools in preoperative psychiatric neurosurgery evaluation.

Disorder	Specific functional aspects to assess	Additional recommended instruments
Tourette syndrome	Social, occupational, and academic interference; impact of tics and comorbidities.	School/workplace reports.
Pathological aggression	Degree of required supervision, institutionalization, need for physical restraints.	Caregiver observations; institutional records.
Substance use disorders	Quality of life, addiction severity, psychosocial and medical impact.	WHOQOL-BREF (102), SF-36 (70), EuropASI (61).
Anorexia nervosa	Self-care, nutritional intake, personal autonomy.	Family/Caregiver Observation

WHOQOL-BREF, World Health Organization Quality of Life – BREF; SF-36, Short Form Health Survey 36; EuropASI, European Addiction Severity Index.

## Future directions and limitations

The integration of functional neurosurgery into psychiatric care is progressing in parallel with rapid advances in neurotechnology, neuroethics, and data science. Future directions include the implementation of multicenter registries and standardized longitudinal protocols, which may facilitate the comparison of outcomes across centers and improve external validity. Advances in machine learning, emerging network-based biomarkers, and digital phenotyping may also contribute to more precise patient characterization and outcome prediction. As the field evolves, emerging neurobiological and connectomic markers may help refine the relationship between circuit dysfunction, clinical phenotypes, and treatment response. However, current evidence remains insufficient to support their routine use for individualized surgical planning, underscoring the continued importance of comprehensive multidimensional assessment as the foundation of clinical decision-making. Moreover, the incorporation of patient-reported outcomes, quality-of-life measures, and qualitative neuroethical frameworks is essential to capture the subjective impact of psychosurgical interventions and support shared decision-making. As these innovations mature, they may foster a more personalized, ethically grounded, and internationally harmonized approach to psychiatric neurosurgery.

As a narrative review, this work is inherently limited by its non-systematic methodology, which prioritizes conceptual synthesis over comprehensive literature retrieval. Consequently, there is a potential for selection bias in the sources included. Accordingly, the proposed eligibility criteria and assessment domains should not be interpreted as empirically validated tools or evidence-graded recommendations. Rather, they represent a pragmatic clinical synthesis intended to support assessment planning, multidimensional evaluation, and longitudinal outcome monitoring. In addition, while obsessive–compulsive disorder and depression are supported by relatively robust data, other indications such as pathological aggression, substance use disorders, schizophrenia, and anorexia nervosa are still in early investigative phases, often based on case series or compassionate-use reports. This variability highlights the need for controlled, prospective, multicenter studies with standardized protocols and long-term follow-up to consolidate the evidence base. Furthermore, cultural, regulatory, and resource differences across regions may influence the feasibility and applicability of proposed frameworks.

Future work should include indication-specific systematic reviews, expert consensus initiatives, and prospective multicenter validation studies to determine which assessment domains are most predictive of safety, clinical response, and long-term functional outcome.

## Conclusions

Functional psychosurgery has transitioned from a marginal and ethically contentious practice to an emerging, clinically viable option for selected patients with severe, treatment-refractory psychiatric disorders. When applied within specialized centers and under rigorous ethical, clinical, and regulatory oversight, these interventions can yield substantial symptom relief, restore functional capacity, and improve quality of life in individuals who have exhausted standard therapeutic avenues.

The principal contribution of this review is the proposal of a circuit-informed framework for multidimensional preoperative assessment. By linking disorder-specific neurobiological dysfunction to relevant psychopathological, cognitive, and functional domains, this approach may facilitate more comprehensive patient characterization and more meaningful postoperative outcome monitoring across psychiatric indications. This framework should be interpreted as an adjunct to, rather than a replacement for, multidisciplinary expert judgment, formal consensus recommendations, ethics committee review, and applicable local regulatory requirements.

At present, the primary contribution of circuit-informed assessment is not the identification of optimal surgical targets, but the systematic characterization of the psychopathological, cognitive, and functional dimensions most relevant to each disorder and their longitudinal monitoring following intervention.

Nevertheless, the field must advance with scientific rigor, ethical caution, and sustained interdisciplinary collaboration. Strengthening the empirical foundation of psychiatric neurosurgery will require harmonized protocols, international registries, and patient-centered outcome measures, ensuring that innovation remains aligned with patient safety, autonomy, and long-term benefit.

Rather than focusing exclusively on diagnostic categories or surgical targets, future assessment frameworks should aim to capture the psychopathological, cognitive, and functional dimensions most closely linked to the neural circuits implicated in each disorder.

## Glossary

AEMPS: Spanish Agency of Medicines and Medical Devices
ADHD: Attention-Deficit/Hyperactivity Disorder
AN: Anorexia Nervosa
APA: American Psychiatric Association
AUQ: Alcohol Urge Questionnaire
BAI: Beck Anxiety Inventory
BDI-II: Beck Depression Inventory-II
BMI: Body Mass Index
BNSS: Brief Negative Symptom Scale
CBIT: Comprehensive Behavioral Intervention for Tics
CBT: Cognitive-Behavioral Therapy
CDSS: Calgary Depression Scale for Schizophrenia
CEIm: Research Ethics Committee
CGI-I: Clinical Global Impression – Improvement
CGI-S: Clinical Global Impression – Severity
CIA: Clinical Impairment Assessment
CM-Pf: Centromedian–Parafascicular Complex
CPT: Continuous Performance Test
CVLT: California Verbal Learning Test
DBS: Deep Brain Stimulation
DTI: Diffusion Tensor Imaging
DSM-5: Diagnostic and Statistical Manual of Mental Disorders, Fifth Edition
EDE-Q: Eating Disorder Examination Questionnaire
EuropASI: European Addiction Severity Index
fMRI: Functional Magnetic Resonance Imaging
FCSRT: Free and Cued Selective Reminding Test
FDA: U.S. Food and Drug Administration
GPi: Globus Pallidus Internus
GTS-QOL: Gilles de la Tourette Syndrome – Quality of Life Scale
HDE: Humanitarian Device Exemption
HDRS: Hamilton Depression Rating Scale
HCQ: Heroin Craving Questionnaire
HRT: Habit Reversal Training
ICD-11: International Classification of Diseases, 11^th^ Revision
ICAP: Inventory for Client and Agency Planning
IDE: Investigational Device Exemption
ITP: Inferior Thalamic Peduncle
LSAS: Liebowitz Social Anxiety Scale
MADRS: Montgomery–Åsberg Depression Rating Scale
MFB: Medial Forebrain Bundle
MOAS: Modified Overt Aggression Scale
MRI: Magnetic Resonance Imaging
NAc: Nucleus Accumbens
OAS: Overt Aggression Scale
OCD: Obsessive–Compulsive Disorder
OCDUS: Obsessive Compulsive Drug Use Scale
PA: Pathological Aggression
PANSS: Positive and Negative Syndrome Scale
PMH: Posteromedial Hypothalamus
ROCF: Rey–Osterrieth Complex Figure
RPM: Raven’s Progressive Matrices
rs-fMRI: Resting-State Functional MRI
SCZ: Schizophrenia
SDS: Sheehan Disability Scale
sgACC: Subgenual Anterior Cingulate Cortex
SNpr: Substantia Nigra Pars Reticulata
STN: Subthalamic Nucleus
SUD: Substance Use Disorder
TLFB: Timeline Follow-Back
TMT: Trail Making Test
TRD: Treatment Resistant Depression
TS: Tourette Syndrome
VTA: Ventral Tegmental Area
VAS: Visual Analog Scale
VC/VS: Ventral Capsule/Ventral Striatum
WHODAS 2.0: WHO Disability Assessment Schedule 2.0
Y-BOCS: Yale–Brown Obsessive Compulsive Scale
YGTSS: Yale Global Tic Severity Scale
